# “I’m Going to Tell You Something I Never Told Anyone”: Ethics- and Trauma-Informed Challenges of Implementing a Research Protocol with Syrian Refugees

**DOI:** 10.3390/ijerph20021261

**Published:** 2023-01-10

**Authors:** Lisa Matos, Monica J. Indart, Crystal L. Park, Isabel Leal

**Affiliations:** 1 William James Center for Research, ISPA—Instituto Universitário, 1149-041 Lisbon, Portugal; 2 Graduate School of Applied and Professional Psychology, Rutgers University, New Brunswick, NJ 08901, USA; 3 Department of Psychological Sciences, University of Connecticut, Storrs, CT 06269, USA

**Keywords:** ethics-in-practice, vicarious trauma, vicarious growth, compassion satisfaction, Syrian refugees, cross-cultural research, transcultural practice, complex trauma

## Abstract

As research subjects, refugees have numerous potential vulnerabilities. This study aimed to examine the ethics- and trauma-informed challenges of implementing a mental health research protocol with Syrian refugees living in Portugal. Guided by the integrated meaning-making model, the research project “Journeys in Meaning” employed a mixed-methods cross-sectional design to explore posttraumatic cognitive processing in refugees using two phases of data collection: two focus groups (Phase 1) to test the protocol and 39 in-depth individual interviews (Phase 2) to implement the protocol. Results examine the strategies used to address the following: methodological challenges related to protocol design, participant recruitment, and language; ethics- and trauma-informed challenges aimed at minimizing harm and maximizing benefit to participants that followed social justice principles; and perceived compassion fatigue on the part of the researcher following repeated empathetic exposure to traumatic content. Findings suggest the need for adaptive approaches to research with refugee populations that challenge strict compliance with the traditional principles of “do no harm” and researcher neutrality, and that accommodate individual and community complexities.

## 1. Introduction

Over the last decade, more than 100 million people were forcibly displaced from their homes [1]. A minority of these refugees found safety in Western countries of asylum following exposure to debilitating traumatic events and devastating losses [2]. In post-migration settings, refugees remain vulnerable to significant daily stressors related to poverty, discrimination, language, and cultural adaptation, which can be aggravated if they have unclear or non-permanent legal status, if their families are at risk, or if they depend on state-sponsored host programs [3]. For host countries, sudden increases in refugee arrivals pose significant economic, social, and public health challenges. These challenges call for data-driven policies that promote psychological well-being as a condition for successful long-term integration [4,5].

The Syrian war has led to the forced displacement of an estimated 12 million civilians since its onset in 2011 [1]. Studies have thus far documented the negative mental health effects of the war, including posttraumatic stress disorder (PTSD), often co-morbid with depression and anxiety [6,7], which can impair refugees’ ability to learn new skills and rebuild their lives [8]. However, despite the severity of trauma, refugees also appear to experience positive psychological adjustment and perceive growth in the aftermath of trauma [9].

The search for meaning in the aftermath of trauma is a critical step in the process of posttraumatic recovery [10]. The trauma recovery literature posits that events that challenge individuals’ orienting systems require cognitive reappraisal efforts through meaning-making processes to rebuild shattered assumptions about the world (e.g., expectations of safety, justice, or self-reliance) and reduce distress [11]. When successful, meaning-making can lead to a return to pre-trauma psychological functioning, as individuals change their appraisal of the potentially traumatic event (PTE) to fit their global meaning (i.e., their core beliefs, life goals, and sense of purpose) or to perceived positive life changes [11]. Although refugee trauma can be severe enough to shatter core beliefs (which is necessary to initiate the process of searching for meaning), current meaning-making theoretical frameworks remain largely informed by Western perspectives, predominantly focus on single-event, personal disruptions, and conceptualize meaning-making as an individual process [12]. Despite recent studies with refugee populations that examine important aspects of meaning-making (e.g., meaning-making across generations [13] or through narrative methods [14]), it remains unclear how cumulative, collective traumatic experiences affect refugees’ integrated meaning-making experience, its determinants and outcomes, as well as impact on post-displacement psychological well-being. Studies that address this gap in empirical knowledge are crucial to inform psychological growth-promoting interventions with forcibly displaced populations.

As research participants, refugees present intrinsic and extrinsic vulnerabilities that make them especially susceptible to harm and exploitation [15]. To protect refugees from emotional distress as they revisit details of overwhelming events [16], ethics committees often act as gatekeepers that, however well-intended, may establish unreasonable safeguards [17] that can further disenfranchise refugees and reinforce patterns of oppression and silence [18]. Although the risks of retraumatization should not be minimized, the distress associated with participating in trauma research has been found to be largely mild, transitory, offset by the benefits of enrollment, and only reported by a minority of participants [19]. Survivors see value in contributing to science-based knowledge that may help others, and, where narrative methods are used, participation can offer an empowering opportunity for individuals to regain control over their life stories and promote agency and healing [20]. Ethically accountable trauma research therefore requires a delicate balance between harm minimization and benefit maximization [18].

In research with refugee communities, strict compliance with fixed ethical principles may set unrealistic expectations and place undue burden on researchers who have to weigh issues of agency, power, language, culture, and distress throughout all phases of study design and implementation [17,20]. When designing and implementing study protocols, refugee trauma researchers are required to integrate ethics guidelines, rigorous methodology, and language and cultural competency, while also being exposed to vast quantities of traumatic material, often with inadequate supervision [16].

The effects of secondary exposure to trauma content on direct service providers, including clinicians, therapists, and humanitarian workers, have been widely documented in the vicarious trauma literature [21,22], yet little is known about its impact on mental health trauma researchers. As some of the adverse consequences of their work, trauma workers can experience secondary traumatic stress, thus displaying symptoms that mirror those of the client; vicarious trauma secondary to shattered worldviews; and compassion fatigue, which entails the loss of ability to empathize with the survivor [23]. However, they can also perceive psychological benefits that include perceived vicarious posttraumatic growth through positive changes in cognitive perspectives, vicarious resilience, and compassion satisfaction [24,25].

As ethics and research committees focus their attention on the potential harm to participants, mental health trauma researchers, who repeatedly and empathically guide individuals through narratives of untold losses and suffering and witness distress firsthand, appear to overwhelmingly be left without a support system [26]. Additionally, at its core, academic work with trauma survivors engages two potentially conflicting and psychologically demanding tasks: on the one hand, meticulous data collection and processing that requires highly analytical and cognitively intense skills, while on the other hand, the ability to empathetically bear witness to the survivor’s experience, which requires employment of emotional skills [27,28].

### Rationale for the Study

In the aftermath of the 2015 surge in Mediterranean crossings, refugee arrivals to Portugal increased significantly, requiring an unprecedented effort by national and local authorities across the country to host arriving communities [29]. With Syrians comprising one of the largest arriving communities, in 2017, we designed a research project, “Journeys in Meaning” (JiM), to assess cognitive restructuring processes in war-exposed Syrian refugees. Findings from the study will inform evidence-based psychological growth-promoting policy and practice with resettled refugees.

Given the compounded vulnerabilities of refugee populations in post-displacement settings, this case analysis aimed to examine the ethics- and trauma-informed challenges of implementing JiM’s Arabic-language protocol as well as the impact of project implementation on the lead researcher.

## 2. Materials and Methods

### 2.1. Study Design

Guided by Park’s integrated meaning-making model [11], the cross-sectional mixed-methods research project relied on qualitative methodology to elicit exploratory research on pre- and post-displacement meaning systems and meaning-making trajectories, while standardized self-report questionnaires were used to assess exposure to PTEs, trauma-related distress, and extent of belief and goal violations. The mixed-methods design allows data triangulation and complementarity, and has been deemed appropriate to capture the complexity of mental health issues in refugees [30]. JiM’s principal research objectives were to: (1) examine exposure to PTEs and associated psychological distress; (2) assess violations of pre-war assumptions; (3) explore narrative accounts of traumatic experiences and subsequent processes of searching for meaning; (4) identify cognitive processes that facilitate or impede meaning-making; and (5) analyze the contribution of refugees’ meaning-making strategies to psychological adjustment. With this research, we expected to identify collectively- and culturally-informed meaning-making processes that would indicate specific needs in Syrians’ post-displacement experience. We posited that: different types of PTEs would violate different meaning systems; not all attempts to find meaning would be growth-promoting; and refugees with completed meaning-making journeys would perceive improved psychological functioning.

The proposed design comprised two phases of data collection. Phase 1, implemented between September and December 2018, would consist of four focus groups (FG) in Lisbon, with 5–7 participants each, organized by gender. FGs would provide an opportunity to test the protocol’s face validity and reach a shared understanding of terminology, including appropriate probing questions [31]. Building on FG findings [32], between January and April 2019, 30 additional refugees living across continental Portugal would participate in cognitive interviews (In-Depth Individual, IDI) to capture detailed accounts of their integrated meaning-making experiences.

JiM was hosted by ISPA—Instituto Universitário’s William James Center for Research and coordinated by the first author, a clinical psychology PhD proponent (hereinafter, the “Researcher”), under the supervision of a Research Committee (RC) and in consultation with an Expert Committee (EC). The RC included the author of the meaning-making theoretical model as well as one refugee trauma and one psychological adjustment scholar. JiM involved strategic partnerships with refugee community leaders, resettlement and community organizations, cultural mediators, and key stakeholders across Portugal, who formed the EC and counseled on cultural, language, outreach, and logistics during project design and implementation. JiM was funded by the Portuguese Foundation for Science and Technology (SFRH/BD/129602/2017 and UIDB/04810/2020) and, prior to interacting with participants, the project received ethical approval of ISPA—Instituto Universitário’s Ethics Committee (Ref. D/004/09/2018) in September 2018.

#### 2.1.1. Participants and Procedures

Eligible participants were recently arrived (≥6 months) Syrian Arabic-speaking adults (≥18 yo). Recruitment included study information sessions hosted by local community and resettlement organizations; distribution of flyers in Arabic, English, and Portuguese to organizations and key stakeholders for affixation and dissemination; and social media postings. Snowball sampling was subsequently used. Study participants signed consent forms and were ensured of confidentiality and anonymity, briefed on potential symptoms and normal reactions to the retelling of their stories, and informed of the possibility of withdrawing at any time and of being referred to psychosocial support as needed. All interviews were audio-recorded following written and oral confirmation of consent.

#### 2.1.2. Materials

The protocol consisted of six documents: (1) Information to participants to be provided at the beginning of the FG/IDI in print and reviewed orally, with a description of the study procedures, research team, clarification of the role of researcher, overview of participant’s rights, confidentiality, risks, and benefits; (2) Consent form to be signed and dated by participants; (3) Socio-demographic questionnaire built for the purposes of the study to collect key determinants of refugee health [5]; (4) Harvard Trauma Questionnaire—Arabic version (*HTQ*; [33]) to assess exposure to trauma events (Part 1; 45 items), torture history (Part 5; 34 items), and trauma symptoms in the two weeks prior to the interview (Part 4); (5) Global Meaning Violations Scale (*GMVS*; [34]) measuring disruption of beliefs (five items) and goals (eight items) in the aftermath of a traumatic event on a five-point Likert scale to be cross-culturally adapted for use with Arabic-speaking refugees; and (6) Semi-structured interview guide to explore pre- and post-traumatic meaning cognitions. The latter established “the events that led you to leave your country” as a baseline to reflect on pre- and post-trauma beliefs, life goals, and sense of purpose. Study materials were designed in English and subsequently translated and back-translated to Arabic.

#### 2.1.3. Data Processing

Participant anonymity was ensured by assigning a numerical code to each participant, which was then used to identify them across all data. Detailed notes on individual reactions and expressed immediate needs or concerns both during and after the interview were kept in a separate tracker. Audio recordings were transcribed, the original files subsequently destroyed, and identifying information removed from transcriptions. Digital files were kept in a separate server, and paper files were placed in a locked cabinet. Only the Researcher had full access to the restricted files, and research assistants signed confidentiality agreements for processing separate datasets.

#### 2.1.4. Expected Outcomes

Expected key outcomes were quantitative (pre-flight PTEs, PTSD diagnosis, extent of belief and goal violations) and qualitative (meanings made of trauma and perceived changes to psychological functioning). Secondary outcomes included preliminary validation of the GMVS-ArabV and integrated pathways of Syrians’ meaning-making processes.

### 2.2. Study Implementation

Data collection effectively started in October 2018 and ended the following May. The Researcher, who was assisted by Arabic language interpreters in Phase 1 FGs, collected all data.

#### 2.2.1. Phase 1: Focus Groups

Six Syrian resettled (*n* = 2) and relocated (*n* = 4) refugees, including five women and one man, signed up for two small FGs in October and December 2018 in Lisbon, following in-person information sessions facilitated by the Researcher. One woman failed to show up; therefore, the final group composition was FG1, *n* = 2 women, and FG2, *n* = 2 women and *n* = 1 man. Participants averaged 37.4 years in age (*SD* = 12.2), spent a mean of 27.8 months in transit (*SD* = 18.8), had been living in Portugal for over two years (*M* = 27.8 months; *SD* = 7.3), and all travelled with their children. The highest level of formal education was *n* = 1 basic, *n* = 1 middle, and *n* = 3 secondary school.

#### 2.2.2. Phase 2: In-Depth Individual Interviews

Twenty-one men (55.6%) and nineteen women (44.4%) between the ages of 19 and 55 (*M* = 27.8; *SD* = 6.5) enrolled in Phase 2. One man dropped-out after becoming distressed during the study. The final sample thus consisted of 39 Syrian nationals: 31 (80%) were beneficiaries of higher education programs for refugees (i.e., “the other one percent”, according to the UNHCR [35]), 3 were spontaneous asylum-seekers, and 5 were EU-relocated refugees. Participants averaged fewer than three years in Portugal (*M* = 33.2 months; *SD* = 19.6) and had exceptionally high formal education: 2 doctoral, 11 Master’s, and 19 Bachelor’s degrees. Seven interviews were conducted in Portuguese, while the remainder was conducted in English. Interviews lasted on average 90 min. All study materials were available in Arabic, English, and Portuguese to ensure consistency of language. Researcher and participants met in partner organizations’ offices or in quiet places of participants’ convenience, and interviews were held in the districts of Braga and Oporto in the north (40%), Aveiro and Coimbra (22%) as well as Lisbon (36%) in central Portugal, and in the southern district of Évora (2%).

#### 2.2.3. Preliminary Results and Dissemination of Findings

Preliminary JiM findings have thus far suggested that: (1) regardless of immigration status, war-affected civilians are exposed to numerous, extreme PTEs as well as to significant pre- and post-migration daily stressors that are also capable of violating meaning systems and thus be perceived as traumatic; (2) refugees make meaning of shattered cognitions throughout their migration journeys, engaging in repeated and cumulative meaning-making trajectories with no definite end; and (3) meanings made of trauma can contribute concurrently to positive and negative psychological adjustment. Preliminary findings also challenge the concept of recovery from trauma as an end state. Principal strategies for dissemination of findings have included presentations in scientific conferences, publications in peer-reviewed journals, and social media postings with Arabic-language abstracts available to allow wider audiences to monitor study progress. Key stakeholders (e.g., members of the Portuguese parliament, local government representatives, and journalists) and individual study participants are also informed of new publications.

## 3. Results

### 3.1. Strategies to Address Methodological Challenges

#### 3.1.1. Protocol

The integrated meaning-making model [11] was originally developed and tested largely in US-based student populations. Its applicability to survivors of refugee trauma thus required preliminary consultations with key Syrian community members and scholars to assess if and how meaning-making and its derivative processes would be similarly understood in the target population [36]. Although no culture-informed disparities were found, constructs such as “life as meaningful,” “core beliefs,” or “sense of purpose” were flagged as potentially complex for prospective participants to readily apprehend. As such, two strategies were devised to maximize suitability of the approach and language. On the one hand, we employed a Syrian translator with active field experience with arriving communities, who counseled on terminology and the best approaches based on the target population’s perceived ability to grasp concepts and disclose personal experiences. On the other hand, the RC recommended that the protocol be pilot tested in focus groups before proceeding with cognitive interviews and that time be reserved at the end for participants to provide feedback on content and language. Phase 1 FG participants found the protocol appropriate and the concepts and wording familiar, albeit “difficult” to reflect upon.

Subsequent to FG findings, adjustments to language included: “worldviews/how you see the world” being employed to convey “global meaning,” similarly to “what you lived for before the war/live for now” to access “sense of purpose.” The protocol further anticipated strategies to help ground participants and guide them away from the implicit abstraction of some constructs by providing concrete examples (e.g., going from uncued questions to cued probing) and setting a baseline for potential disruptions by thinking back on participants’ “own” lives to connect them to their concrete experiences instead of offering general impressions.

#### 3.1.2. Recruitment

To minimize potential exploitation of particularly vulnerable individuals [37], we relied on mediation through resettlement agencies and community leaders to help build trust between eligible participants and the Researcher [18]. This included information sessions about the study held in the context of a group activity (e.g., at the end of the agency’s monthly meeting with beneficiaries) where the Researcher was invited to present the study and introduced to prospective participants by individuals with whom they had an ongoing relationship. Despite these efforts, Phase 1 yielded only five participants who self-organized to integrate two small FGs. We expected FGs to encourage participation in potentially stigmatizing mental health research [38]. Instead, the group setting proved to be a deterrent to enrollment. Community research fatigue, concerns about confidentiality, and preoccupation with immediate socioeconomic needs were suggested as reasons why prospective participants may have been reticent to participate. Considering the confidentiality concerns expressed by participants during the test phase, a decision was made to eliminate language interpretation in Phase 2, effectively requiring participants to be fluent in English or Portuguese.

Participant enrollment in IDIs was successful to the point of drawing a waitlist beyond data saturation and logistical and programmatic ability to accommodate additional participants. Keys to successful recruitment were: the individual setting; elimination of interpreters, who would have likely been from the same community; elimination of inclusion criterion requiring refugee status, as to capture a diversity of refugee experiences, regardless of legal status or pathways to safety; and snowball recruitment by individuals who participated in the study and subsequently recruited and in many cases helped organize interviews with other participants in their towns.

#### 3.1.3. Language

There were several challenges pertaining to language, common to field conditions, but challenging for scientific accuracy. The main challenge pertained to Phase 2 participants being required to access or express certain ideas or constructs in a non-native language [39]. However, the importance of giving participants an opportunity to speak freely cannot be overstated, especially in a community where social identity had been shattered by the war [40]. Feelings of mistrust and isolation are not uncommon in the aftermath of the collective trauma [41], and they were painfully articulated by several participants, one of whom pointedly asked, “do you think my [Syrian] friends would tell me the things they told you?”

#### 3.1.4. Outputs of Narrative Methods

There were marked differences in Phase 1 and Phase 2 participants’ abilities to access and narrate posttraumatic cognitions. FG participants, all in Portugal on refugee status, appeared generally unavailable to engage in abstract, deep reflections about meaning in life, and they often rerouted the discussion to more pressing socioeconomic needs. It is unclear how the group setting, issues of confidentiality, and level of formal education impacted individual ability to delve into meaning-making narratives. In Phase 2, participants were largely beneficiaries of higher education programs for refugees and appeared to be cognitively better equipped to engage in meaningful reflection. Additionally, regardless of past trauma and significant daily stressors, student-refugees were generally able to set aside discussions on potential immediate concerns, access the invoked cognitions, and provide rich data.

### 3.2. Strategies to Address Ethics- and Trauma-Informed Challenges

#### 3.2.1. Harm Minimization

The protocol included safeguards to address distress resulting from, and subsequent to, the research encounter, which were employed as appropriate to individual needs. In addition to explaining the nature of symptoms associated with revisiting traumatic experiences and referral to psychosocial support in the community, other safeguards included destigmatization of help-seeking, empathetic expression of sorrow, recognition of strength and resilience, and offering strategies to minimize distress (e.g., breathing and grounding exercises). The latter was especially useful to regroup FG2 participants and the interpreter following a particularly difficult account, in Arabic, that led to collective crying and to the Syrian interpreter also becoming visibly agitated and momentarily unable to convey to the Researcher what had been narrated. In Phase 2, trauma content-related distress had various manifestations, the most common of which included participants becoming emotional subsequent to, or avoidant at the prospect of, retelling a specific event. As such, three participants opted for writing down instead of verbalizing a difficult occurrence and then asked the Researcher to read the description (e.g., one young woman wrote, “in 2013, I was blown-up with my friends when I was in school. I saw many friends dead”). At least two participants reported pre-interview anxiety leading to severe insomnia prior to the interview. Lastly, one torture survivor was despondent throughout the research encounter, leading the Researcher to end the interview early, rather than attempting to probe, and to focus on discussing options of care.

#### 3.2.2. Benefit Maximization

All study participants were offered a EUR 10 voucher. Phase 1 recruitment materials included reference to the voucher, which was used as incentive for enrollment. However, following recommendations from FG participants, who found the amount insufficient to serve as an incentive, in Phase 2, reference to the voucher was removed from the materials, and when it was gifted at the end of the interview, it was overwhelmingly welcomed as a pleasant surprise.

Numerous participants expressed being thankful for the opportunity to share their stories and, especially among student-refugees, to contribute to evidence-based knowledge that might help their community. Some participants further expressed their gratitude by offering to help recruit others, effectively becoming project champions. To some, the research encounter also presented an important opportunity for healing. One rare and powerful healing experience occurred when a young woman became emotional after reading the trauma symptoms in the HTQ. As she wiped away the tears, she stated: “until now—until just now!—I blamed [life in] Portugal for my suffering. Now I understand it’s normal.” Lastly, on follow-up, four participants expressed the relief they felt for “letting things out,” being “able to say things that [they] kept to [them]sel[ves] for a long time,” and feeling “much better after talking to [the Researcher];” most tellingly, especially among a community that had been receiving so much attention from Portuguese researchers, civil society, the media, and government institutions alike, one participant sent a post-interview message stating, “we rarely find a person who asks and listens. So I enjoyed that.”

### 3.3. Impact of Refugee Trauma Research on the Researcher

#### 3.3.1. Researchers as Agents of Social Justice

The protocol included strategies to counter participant fragility and experienced injustices as well as to foster strength and dignity [20]. Although refugee health researchers are in unique positions to detect and possibly alert service providers to needs disclosed during the research encounter, striking a healthy balance between neutrality and rigor and consequential thinking (i.e., anticipating and weighing harm and benefit [18]) can be mentally taxing. Additionally, while research widely differs from service provision, the distinctions may appear unfair and incomprehensible to those ineligible to participate [15] as well as overwhelming to researchers if confronted with misguided participant expectations.

Some of the harm minimization and benefit maximization strategies followed social justice principles [42,43]. These strategies included: the empathetic expression of sorrow and outrage; appreciation for individual, family, and community resilience; holding someone’s silence when words were insufficient to describe their suffering; or simply bearing witness to one’s story. The protocol subsequently included individual follow-up to thank each participant for their participation, ask about their well-being after revisiting such difficult personal experiences, and inquire about the issue that was most pressing to them (e.g., referral to legal services for a stateless Palestinian refugee, asking about a sick parent, or connecting a participant to a local basketball team).

#### 3.3.2. Fatigue and Trauma Exposure

Language barriers, scheduling logistics, difficulty accessing the complex cognitions that were the object of the study, repeated empathetic engagement, and exposure to trauma content were all issues that put the Researcher at risk for psychological distress [44]. In the early stages of data collection, access to individual participants’ meaning systems was, at best, challenging, with marked improvement in study outputs with the enrollment of student-refugees. Although students reported having never given much thought to the meaning of life, they largely and more promptly welcomed the discussion and were available to explore their complex cognitive processing.

In terms of the logistics of interviewing, there was an initial concern not to schedule more than three IDIs in a given week to give the Researcher time to process the material and create room for appropriate, empathetic witnessing [28] before the next interview. However, as the pace of enrollment increased and eventually peaked at four back-to-back interviews per day, in addition to feeling emotionally depleted, the Researcher began feeling guilty for no longer being able to hold each individual account with the space and respect it merited. The Researcher also noted progressively decreased ability to express empathy and began feeling emotionally numb towards the end of data collection, which studies have found to be protective [45]. Having surpassed data saturation, individual accounts became increasingly similar, and fatigue began to take hold. This phenomenon is evidenced through shorter interjections by the Researcher, as well as missed opportunities to explore themes that had otherwise been comprehensively explored with earlier participants.

#### 3.3.3. Trauma, Survival, and Privilege

“I’m going to tell you something I never told anyone.” Like this 21-year-old man, other participants made similar announcements before sharing frightful, humiliating, or shame-filled experiences. Although the study protocol did not call for the narration of specific PTEs, as such data was intentionally collected quantitatively to minimize distress [19], participants frequently wanted to narrate the extreme experiences that had challenged their meaning systems. On such occasions, the Researcher promptly engaged the protective cognitive strategies she had been trained to employ over 12 years of fieldwork with survivors of torture: listening empathetically while focusing on capturing information relevant to the object of the meeting, debriefing (preferentially) with a refugee trauma colleague, and, if needed, taking a “mental health” day off. Yet, being privy to participants’ narratives gave the Researcher an overwhelming sense of privilege and appreciation for their own as well as their community’s strength and resilience, which has been shown to provide opportunities for psychological growth [46]. This positive reappraisal of worldviews is something the Researcher had experienced earlier in her career, including reappraised sense of purpose, which continued to prove adaptive throughout study implementation.

## 4. Discussion

This study assessed the challenges of implementing an Arabic-language mental health research protocol with Syrian refugees living in a post-migration setting. The complexity of constructs and cognitive processing under study, language and cultural specifications, severe trauma exposure, and the compounded vulnerabilities associated with participants’ recent arrival in Portugal called for a network of advisors that could help bridge the gap between rigorous design and the realities of field research work. Although exacting on the Researcher, the flexibility required to accommodate methodological, ethical, and trauma-informed challenges was manageable due to a combination of prior training (e.g., empathetic engagement and witnessing as a trained skill [27,28]), help-seeking behavior (e.g., request for regular supervision [28]), and the supportive organizational context [47] provided by Research and Expert Committees alike throughout all phases of the project.

The lessons from protocol implementation focus on the need for adaptive approaches to recruitment, practice, agency, harm–benefit balance, and researcher self-care, which render traditional ethics principles that emphasize strict application of the principles of “do no harm” or researcher neutrality at odds with the practical challenges of doing research with forced migrants. Strict compliance with those principles may unintentionally disenfranchise already marginalized population samples [16]. Instead, refugee scholars have discussed the need for a new ethics paradigm that promotes flexibility, ethical as well as ecological thinking, and where researchers have an obligation to bring benefit to participants and to commit to principles of social change [15,17,19].

Although JiM participants occasionally evidenced distress as they recalled particularly difficult life events, the symptoms were not only mild and transitory but also appropriate to content. The protocol included safeguards to offset potential helplessness or loss of control arising from, or subsequent to, the research encounter [19] as well as strategies to provide opportunities for healing and justice, as appropriate. These were achieved through debriefing protocols that normalize distress, promote the empathic expression of outrage, and offer a participant-centered approach that recognizes researcher–participant power differentials and vulnerabilities and strengths as relational [20]. In our research, although follow-up protocols were often time- and emotional-resource-consuming, participants reported feeling thankful, reassured of the purpose of their contribution, and often empowered as they reclaimed control over their life story and, as some shared, found purpose in their suffering (e.g., reaffirmed their commitment to participate in postwar reconstruction). The ability to regain narrative authority over one’s life circumstances and reappraise experiences and worldviews through narrative methods can protect a sense of continuity with a shattered past [48] and has been associated with positive psychological adjustment [49,50]; it can also be an important output of the research encounter.

Given repeated reports during Phase 1 recruitment of research fatigue among the Syrian community in Portugal, as well as shattered community ties that compromised trust both in the group setting and with language interpreters of Arab background, the subsequent recruitment of highly educated participants with multi-language proficiency provided meaningful opportunities for safe disclosure, reflection, and healing, despite the methodological limitation of having individuals discuss complex cognitions in a non-native language. The commitment to renegotiate recruitment based on information gained in previous studies has been found to promote trust between researchers and displaced populations [18] and is consistent with this study’s findings. Lastly, as the Researcher began evidencing signs of compassion fatigue, a decision was made to end data collection, given that data saturation had also been surpassed and despite there still being participants awaiting interviews. It is unclear what the decision would have been had data collection objectives not yet been reached and what strategies would have otherwise been implemented to support the Researcher’s wellbeing.

Although safeguards for participants, including debriefing and follow-up, were thoroughly discussed and incorporated in the protocol, strategies to protect the Researcher from harm were defined and implemented on an ad hoc basis. It is therefore crucial that, in addition to those for participants, protocols also integrate clearly defined safeguards for researchers, with provisions for self-care and regular supervision that, at a minimum, minimize secondary trauma, compassion fatigue, and burnout [23,28] but also offer opportunities for vicarious resilience, growth, and compassion satisfaction.

Refugee scholars have an opportunity to help shape the discourse and public perceptions of refugees. Because forced migrants are inherently political subjects who invoke strong public opinions, centering their experiences on past trauma and vulnerability can contribute to their further stigmatization [5,43]. It is therefore important to include members of the target community throughout the research process in order to achieve a balanced representation of findings that honors the complexity and depth of individual and community experiences.

## 5. Conclusions

Finding the right balance between scientific rigor and the multitude of challenges of designing and implementing mental health research with refugees requires numerous compromises to accommodate individual and community complexities. There is a need for alternative, culturally informed ways of providing consent that empower refugees, recognize existing power imbalances, and offer opportunities for agency and narrative continuity [17,20]. Study protocols should incorporate safeguards to offset any sense of helplessness by participants [19] arising not only during, but *subsequent to*, the research encounter, as well as safeguards to protect researchers’ wellbeing before, during, and after research, namely through trauma-informed training and regular supervision and mentoring. Refugee mental health research that empowers study participants and acknowledges real-world conditions can lead to actionable and effective outcomes that inform host countries’ policies and practice and favorably impact the well-being of arriving communities.

## Data Availability

The datasets generated and analyzed during this study include a large qualitative component and is not publicly available.
